# Corticospinal premotor fibers facilitate complex motor control after stroke

**DOI:** 10.1002/acn3.52159

**Published:** 2024-07-28

**Authors:** Theresa Paul, Matthew Cieslak, Lukas Hensel, Valerie M. Wiemer, Caroline Tscherpel, Christian Grefkes, Scott T. Grafton, Gereon R. Fink, Lukas J. Volz

**Affiliations:** ^1^ Medical Faculty, University of Cologne, and Department of Neurology University Hospital Cologne Cologne Germany; ^2^ Institute of Neuroscience and Medicine Cognitive Neuroscience (INM‐3), Research Centre Juelich Juelich Germany; ^3^ Department of Psychiatry, Perelman School of Medicine University of Pennsylvania Philadelphia Pennsylvania USA; ^4^ Department of Neurology, University Hospital Frankfurt Goethe University Frankfurt Frankfurt am Main Germany; ^5^ Department of Psychological & Brain Sciences University of California Santa Barbara California USA

## Abstract

**Objective:**

The corticospinal tract (CST) is considered the most important motor output pathway comprising fibers from the primary motor cortex (M1) and various premotor areas. Damage to its descending fibers after stroke commonly leads to motor impairment. While premotor areas are thought to critically support motor recovery after stroke, the functional role of their corticospinal output for different aspects of post‐stroke motor control remains poorly understood.

**Methods:**

We assessed the differential role of CST fibers originating from premotor areas and M1 in the control of basal (single‐joint muscle synergies and strength) and complex motor control (involving inter‐joint coordination and visuomotor integration) using a novel diffusion imaging approach in chronic stroke patients.

**Results:**

While M1 sub‐tract anisotropy was positively correlated with basal and complex motor skills, anisotropy of PMd, PMv, and SMA sub‐tracts was exclusively associated with complex motor tasks. Interestingly, patients featuring persistent motor deficits showed an additional positive association between premotor sub‐tract integrity and basal motor control.

**Interpretation:**

While descending M1 output seems to be a prerequisite for any form of upper limb movements, complex motor skills critically depend on output from premotor areas after stroke. The additional involvement of premotor tracts in basal motor control in patients with persistent deficits emphasizes their compensatory capacity in post‐stroke motor control. In summary, our findings highlight the pivotal role of descending corticospinal output from premotor areas for motor control after stroke, which thus serve as prime candidates for future interventions to amplify motor recovery.

## Introduction

Motor impairment after stroke is commonly caused by damage to the corticospinal tract (CST),^1^ which comprises descending fibers originating from the primary motor cortex (M1) as well as various premotor areas.^2^, ^3^ While the increased involvement of premotor regions in motor control after stroke is well established,^4^, ^5^, ^6^ the functional role of CST projections from premotor areas, here referred to as *corticospinal sub‐tracts*, remains largely unclear. Considering that premotor areas contribute a substantial amount of CST fibers descending to the spinal level,^7^ these fibers seem readily situated to facilitate motor control after stroke via descending signals originating from premotor areas. Previous studies analyzing corticospinal sub‐tracts originating from premotor areas^8^, ^9^, ^10^, ^11^, ^12^, ^13^, ^14^, ^15^, ^16^ support this notion yet report inconclusive findings regarding the differential involvement of distinct tracts emerging from ventral and dorsal premotor areas (PMv, PMd) and supplementary motor area (SMA). A possible explanation for this inconsistency lies in the differing quantification of tract integrity across studies. Most studies relied on CST lesion overlaps rather than diffusion MRI (dMRI),^8^, ^10^, ^12^, ^13^, ^14^, ^15^, ^16^ which cannot capture the effects of secondary degeneration of axons passing through the lesion, commonly referred to as *Wallerian degeneration*.^17^, ^18^ Exclusively focusing on lesion information also neglects the motor system's structural reserve, which reflects the brain's premorbid level of structural connectivity enabling motor recovery.^19^

Besides these methodological limitations, the assessment of motor performance varied widely across studies. Given the distinct (patho‐)physiology underlying distinct aspects of motor control post‐stroke,^20^, ^21^ corticospinal output from distinct premotor areas and M1 might differentially contribute to basal and complex motor control after stroke. From an anatomical perspective, tracer studies in macaque monkeys suggest that each premotor area possesses a unique efferent system facilitating specific aspects of motor control.^22^ Thus, the functional roles of descending fibers are likely tied to a region's functional specialization: The SMA is thought to support the execution of sequential movements^23^ as well as the timing^24^, ^25^ and initiation of upper limb movements.^26^ Conversely, PMv and PMd are essential for reaching and grasping movements.^27^ While PMv facilitates the positioning of fingers around objects, PMd supports controlled reaching movements by coordinating the sequential recruitment of muscles, in particular the transition from grasping to manipulating an object.^28^ One might hence expect that a region's compensatory potential arises from its functional specialization, which would predestine premotor areas to support complex hand movements after stroke. Alternatively, premotor areas might more flexibly support various aspects of motor control depending on the stroke‐induced impairment. This would allow them to contribute to a wide range of movements outside their physiological repertoires including basal muscle activation.

To address these questions, we used standardized tests to differentiate basal (simple muscle synergies across a single joint and strength) and complex (requiring inter‐joint coordination and visuomotor integration) upper limb motor control. To overcome previous methodological limitations, integrity of each corticospinal sub‐tract was quantified using a novel compartmentwise analysis approach.^17^, ^29^ We hypothesized that CST fibers emerging from M1 are sufficient to carry out basal motor functions. In contrast, more elaborate motor skills of the paretic hand likely rely on an interplay of descending signals from M1 and premotor areas, particularly PMv and PMd, given their pivotal role in reaching and grasping in healthy individuals.

## Methods

### Sample

Twenty‐five chronic stroke patients (mean age = 66.68, std = 11.25, 20 male, 5 female) formerly hospitalized at the University Hospital Cologne, Department of Neurology (Table S1) and 22 healthy age‐matched controls (mean age = 67.05, std = 6.59, 16 male, 6 female) were included. Inclusion criteria for patients were (1) first‐ever ischemic stroke at least 6 months earlier, (2) unilateral hand motor deficit in the acute post‐stroke phase, and (3) age 40–90 years. Exclusion criteria were (1) contraindications to MRI, (2) cerebral hemorrhage, (3) bihemispheric infarct lesions, (4) re‐infarct or other preexisting neurological diseases, as well as (5) severe aphasia or neglect. All participants provided informed written consent. The study was approved by the ethics committee of the Medical Faculty at the University of Cologne and carried out in accordance with the Declaration of Helsinki. While the current patient cohort was analyzed before,^17^, ^20^ all analyses presented in the present study are novel and there is no overlap with our previous work.

### Behavioral testing

While isolated movements involving only a limited number of muscle groups do not require complex inter‐joint coordination, more complex forms of motor control like object manipulation impose higher demands on the human motor system.^30^ Thus, compensatory strategies employed by the stroke‐afflicted motor system likely vary depending on the level of movement complexity. Unlike previous studies that only focused on one aspect of motor control, we therefore differentially assessed basal and complex motor control in the present study. Complex motor performance representing activities of daily living was assessed via the Action Research Arm Test (ARAT) comprising the subscales grasp, grip, pinch, and gross movements.^31^ The test involves tasks such as picking up wooden blocks, pouring water from one glass to another or lifting up small objects using a pinch grip. Conversely, basal movements were evaluated using the Motricity Index (MI),^32^ which assesses the ability to execute an isolated movement against gravity or resistance, thereby testing simple synergies and muscle strength.^33^ The upper limb scale includes the items of shoulder abduction, elbow flexion, and pinch grip, thereby assessing movement for each joint individually.^34^

### Tract characteristics

**Figure 1:**
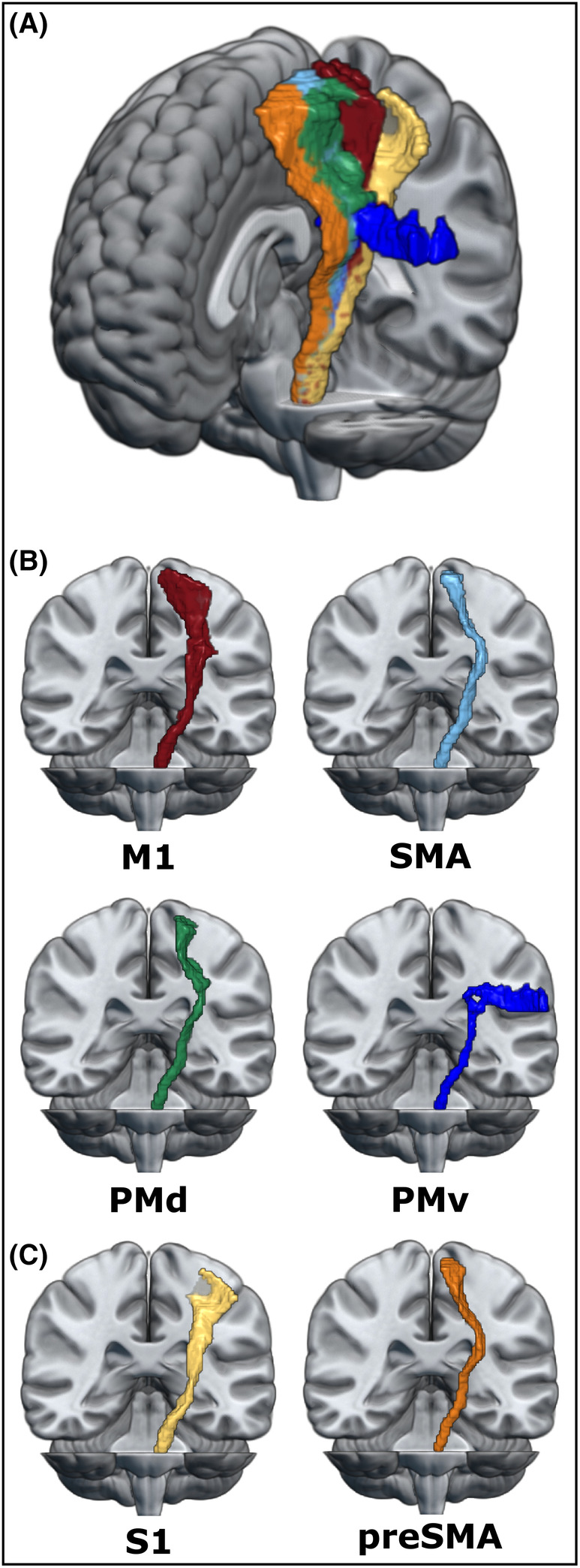
Overview of sensorimotor tracts. (A) Overview of all six sensorimotor tracts as defined in the sensorimotor area tract template (SMATT).^35^ (B) Individual corticospinal sub‐tracts according to their cortical origin. (C) Additional sensorimotor tracts. Images were created in MRIcroGL. M1, primary motor cortex; PMd, dorsal premotor cortex; PMv, ventral premotor cortex; preSMA, pre‐supplementary motor area; S1, primary somatosensory cortex; SMA, supplementary motor area.

Another major limitation of previous studies on premotor CST fibers pertains to the quantification of tract damage. Many previous studies used their own templates for the quantification of lesion overlap or extraction of anisotropy, which oftentimes resulted in a considerable tract overlap of up to 80%.^8^ To increase generalizability and validity, we based our analyses on tract templates with relatively little overlap between tracts that were developed and validated using an independent data set (Fig. 1).^35^ The data set included corticospinal sub‐tracts emerging from M1, PMd, PMv, and SMA as well as two sensorimotor tracts descending from the prefrontal area preSMA and the primary somatosensory cortex (S1). We included these additional tracts as “negative” controls as they feature projections in close proximity to the CST without generating descending motor signals. Of note, while these templates were externally validated and thus helped to limit biases related to determining tract masks during the analysis process, each set of templates comes with its own biases as a result of the underlying sample and the methods used to create them. Future studies might therefore apply alternative approaches to define tract templates.

### Acquisition and preparation of MRI data

Diffusion spectrum imaging (DSI), T1‐weighted, and T2‐weighted data were recorded using a Siemens MAGNETOM Prisma 3T scanner equipped with a 64‐channel head coil (Siemens Medical Solutions, Erlangen, Germany). Image acquisition, the preprocessing workflow including motion and distortion correction in QSIPrep,^36^ and creation of individual gFA maps were performed as described in detail elsewhere.^17^ Each patient's gFA map was normalized and masked in the following way: (i) Individual white matter (WM) masks were used to limit the analysis to WM. (ii) Lesion masks drawn on T2‐weighted images using MRIcron were applied to focus on secondary degeneration by excluding voxels located within the lesion from further analyses. (iii) To focus the analyses on descending fibers WM voxels were compartmentalized according to the number of trackable directions per voxel,^29^ retaining only voxels with exactly one trackable direction for further analyses. CST‐masks were taken from the sensorimotor area tract template (SMATT), which consists of high‐resolution nifti images of different sensorimotor tracts originating from six different cortical regions, namely M1, S1, PMv, PMd, SMA, and preSMA (Fig. 1).^35^

### Statistical analysis of sub‐tracts

To probe whether lesions affected all sub‐tracts to a similar degree, we computed a repeated‐measures ANOVA with the factor tract (levels: M1, SMA, PMd, PMv, preSMA, and S1) and the dependent variable lesion load (i.e., the percentage of tract voxels affected by the lesion). Next, we tested whether lesions resulted in systematically reduced tractwise anisotropy compared to healthy age‐matched controls by computing a mixed ANOVA with the between factor group (levels: patients, controls) and the within factor tract (levels: M1, SMA, PMd, PMv, preSMA, and S1). Moreover, to rule out that potential group differences reflected a general difference in anisotropy between stroke patients and age‐matched controls, we performed a control analysis comparing anisotropy derived from the right superior longitudinal fasciculus (SLF) between both groups using a tract template developed by Yeh and colleagues.^37^

### Tractwise correlations with motor performance

For each subject and sub‐tract, mean gFA values were derived from all one‐directional voxels representing descending motor fibers^17^ (see Table S2 for tractwise anisotropy per subject). Correlations were computed between the resulting tractwise anisotropy and basal and complex motor control, that is, MI‐arm and ARAT scores. To assess whether premotor sub‐tracts contributed differentially to motor control in patients with persistent motor deficits (*N* = 13), we performed additional correlations between basal and complex motor performance and tractwise anisotropy for this subgroup. The persistence of motor deficits was defined as an ARAT score of less than the maximum score of 57 points.^31^ For each analysis, p‐values were false discovery rate (FDR)‐corrected to account for the number of comparisons. To ensure that our results were not driven by specific distribution assumptions, we repeated these analyses using nonparametric permutation tests. Next, we addressed whether sub‐tracts emerging from premotor areas explained behavioral variance independent from M1 by computing partial correlations between behavioral scores and premotor sub‐tracts while controlling for the influence of the M1 sub‐tract. Similarly, we also computed partial correlations to assess the impact of premotor sub‐tract integrity on the correlation between M1 sub‐tract anisotropy and motor behavior.

### Slicewise analyses along each tract

Previous research has commonly calculated CST anisotropy for predefined regions of interest (ROIs) containing densely packed descending fibers.^38^, ^39^ While these ROIs are thought to closely reflect Wallerian degeneration,^38^ using voxels from the entire length of the CST has recently been shown to more closely reflect motor behavior.^17^ Therefore, a compartmentwise approach was employed that utilizes white matter voxels not directly affected by the lesion from the entire length of the CST. As upper CST sections might be influenced by other reorganization processes such as cortical remapping and axonal sprouting, slicewise correlation analyses were performed to assess which sections of each sub‐tract drove the observed correlations between anisotropy and complex motor behavior. Thus, tract‐specific anisotropy was computed per z‐slice along the entire tract length based on one‐directional voxels and subsequently correlated with ARAT scores.

## Results

### Characterization of corticospinal sub‐tracts

**Figure 2:**
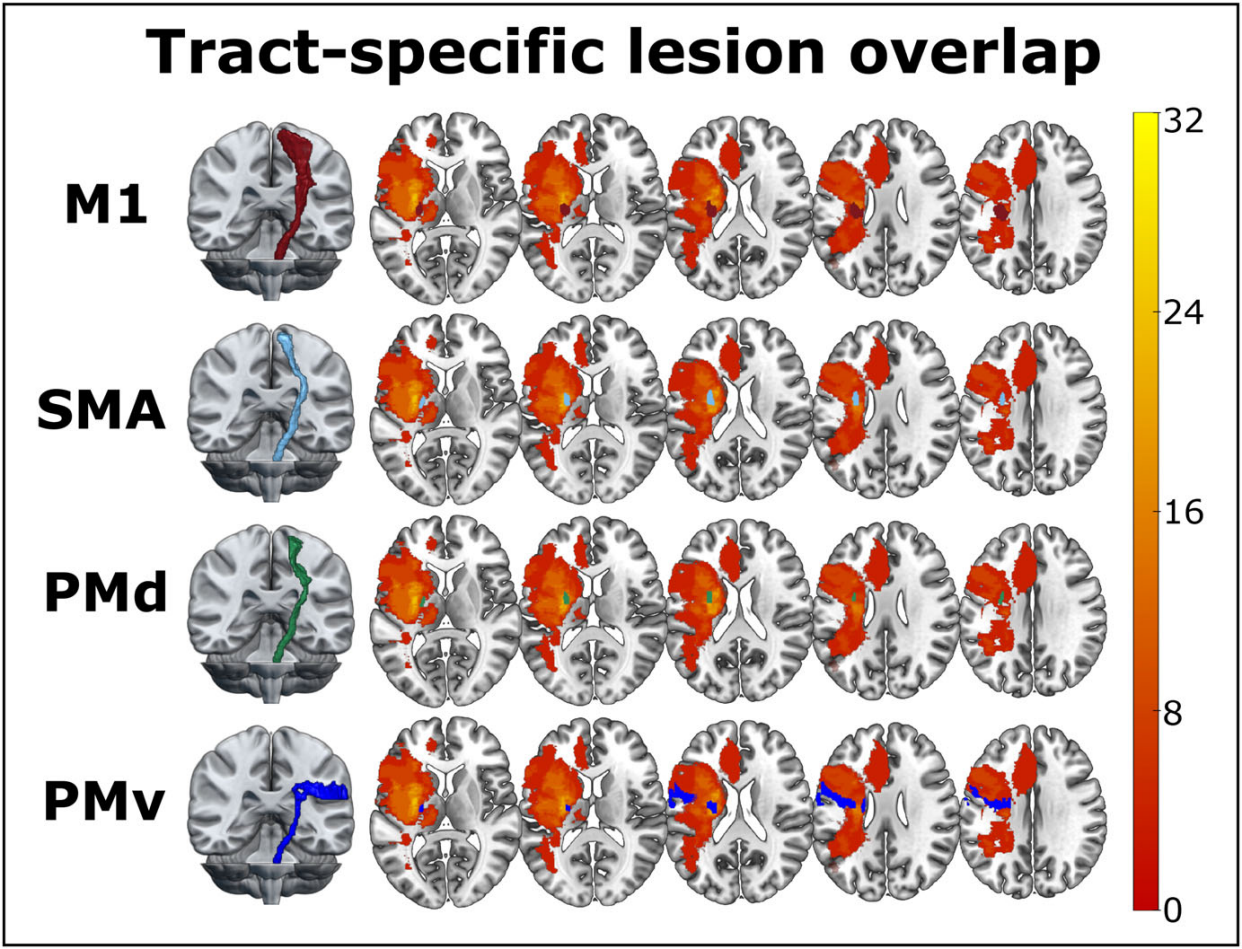
Tract‐specific lesion overlap for corticospinal sub‐tracts descending from distinct motor areas. Lesion overlaps are shown for each corticospinal sub‐tract as a percentage across all subjects. The highest lesion overlap was observed in the posterior limb of the internal capsule (PLIC) with 32% of all patients affected.

According to a repeated‐measures ANOVA, lesion load did not differ significantly across tracts (*F*(2.32, 55.56) = 0.76, *p* = 0.488). Hence, all sub‐tracts were affected to a similar degree across the group. For a tractwise lesion overlap of the descending motor sub‐tracts, please see Figure 2.

Results of a mixed ANOVA showed a significant main effect of group (*F*(1, 45) = 9.39, *p* = 0.004, generalized *η* ^2^ = 0.124) and tract (*F*(2.22, 99.97) = 51.84, *p* < 0.001, generalized *η* ^2^ = 0.270) but no interaction effect (*F*(2.22, 99.97) = 1.32, *p* = 0.270). Post‐hoc t‐tests revealed either a significantly lower (*p* < 0.05 after FDR‐correction) or a statistical trend toward lower anisotropy (*p* < 0.1 after FDR correction) in patients compared to controls for all sub‐tracts confirming that anisotropy was systematically reduced in patients. A Greenhouse–Geisser correction was applied where appropriate. In contrast, anisotropy derived from the right SLF did not differ between groups (*t* (45)= −1.57, *p* = 0.124), indicating that the anisotropy differences were specific to the affected motor system rather than reflecting systematic group differences driven by other factors such as small vessel disease.

### Tractwise correlations with motor behavior

**Figure 3:**
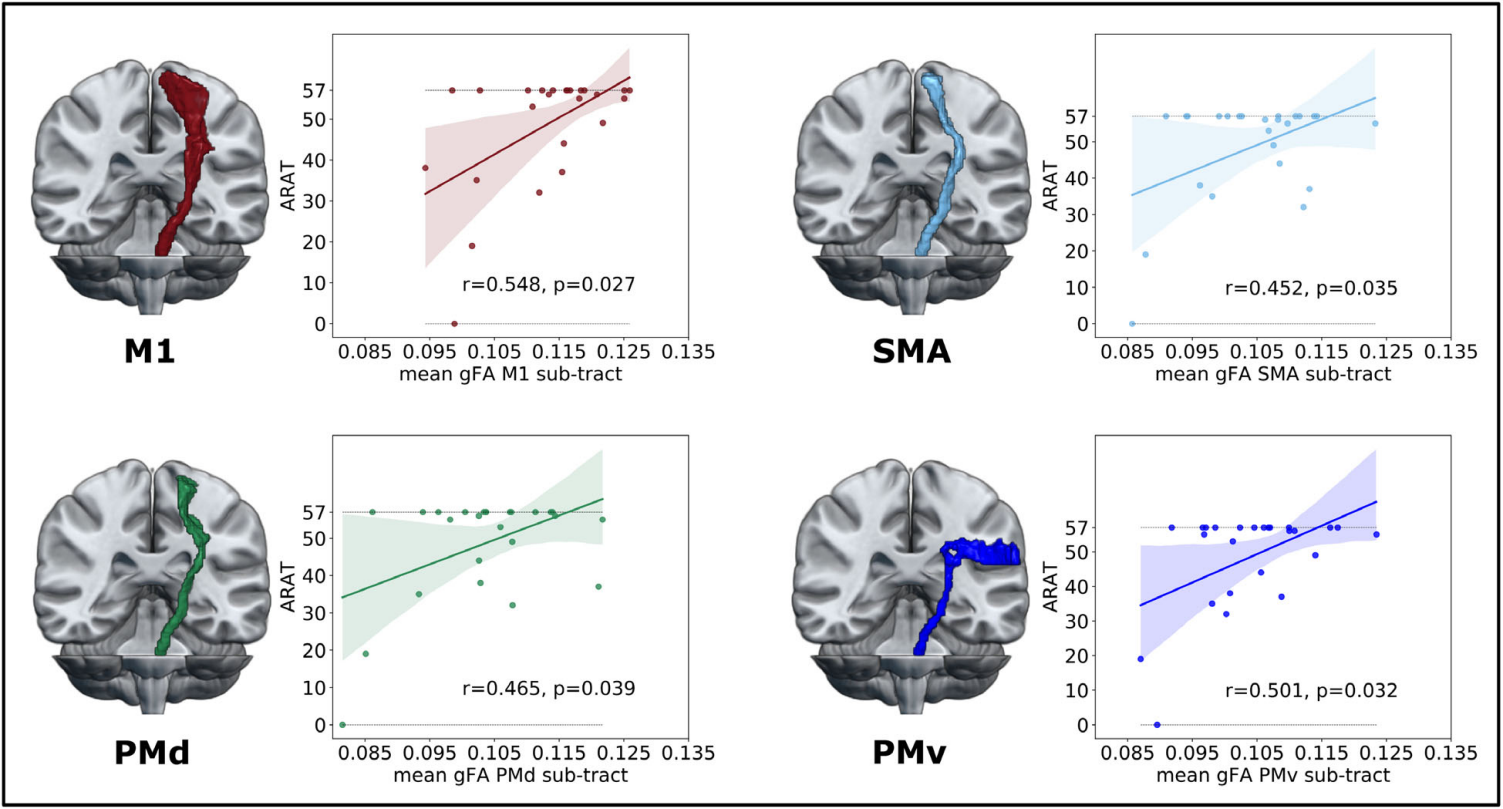
Correlations between anisotropy of corticospinal sub‐tracts and complex motor skills. For each corticospinal sub‐tract, mean gFA was derived from one‐directional voxels along the entire tract length, and correlations with the ARAT score were computed. All reported p‐values are FDR‐corrected for the number of comparisons. M1, primary motor cortex; PMd, dorsal premotor cortex; PMv, ventral premotor cortex; SMA, supplementary motor area.

The MI‐arm score was associated with the M1 sub‐tract but not with sub‐tracts emerging from premotor areas, indicating that the control of individual limb movements was primarily related to M1 output integrity (Table 1). For complex motor skills assessed by the ARAT, we found significant correlations with motor performance for corticospinal sub‐tracts originating from M1, PMd, PMv, and SMA. Thus, our results emphasize the importance of these premotor regions for shaping complex arm and hand movements (Table 1, Fig. 3). In contrast, patients suffering from persistent motor deficits also featured significant correlations between the MI‐arm score and anisotropy of premotor sub‐tracts, suggesting a contribution of premotor areas to basal motor performance exclusively in patients with persistent motor deficits (Table 2). Of note, similar results using nonparametric permutation tests corroborated the robustness of these findings (see Table S3 for details).

**Table 1:** Association between anisotropy and motor control for each corticospinal sub‐tract.

DV	Predictor	*R* ^2^	Pearson *r*	*p*	*p* (FDR)
**ARAT**	**M1**	**0.300**	**0.548**	**0.005**	**0.027**
**ARAT**	**PMd**	**0.216**	**0.465**	**0.019**	**0.039**
**ARAT**	**PMv**	**0.251**	**0.501**	**0.011**	**0.032**
ARAT	S1	0.076	0.276	0.181	0.218
**ARAT**	**SMA**	**0.204**	**0.452**	**0.023**	**0.035**
ARAT	preSMA	0.063	0.250	0.228	0.228
**MI‐arm**	**M1**	**0.316**	**0.562**	**0.003**	**0.021**
MI‐arm	PMd	0.115	0.340	0.097	0.290
MI‐arm	PMv	0.103	0.321	0.118	0.177
MI‐arm	S1	0.073	0.271	0.190	0.228
MI‐arm	SMA	0.107	0.327	0.111	0.221
MI‐arm	preSMA	0.014	0.116	0.580	0.580

Pearson correlations between mean gFA derived from one‐directional voxels and motor behavior for each corticospinal sub‐tract. Bold font indicates significance after FDR‐correction.

**Table 2:** Results for patients with persistent motor deficits.

DV	Predictor	*R* ^2^	Pearson *r*	*p*	*p* (FDR)
**ARAT**	**M1**	**0.504**	**0.710**	**0.007**	**0.009**
**ARAT**	**PMd**	**0.460**	**0.679**	**0.011**	**0.011**
**ARAT**	**PMv**	**0.513**	**0.716**	**0.006**	**0.012**
**ARAT**	**SMA**	**0.571**	**0.756**	**0.003**	**0.011**
**MI‐arm**	**M1**	**0.354**	**0.595**	**0.032**	**0.043**
**MI‐arm**	**PMd**	**0.421**	**0.649**	**0.016**	**0.033**
**MI‐arm**	**PMv**	**0.325**	**0.570**	**0.042**	**0.042**
**MI‐arm**	**SMA**	**0.477**	**0.691**	**0.009**	**0.036**

Tractwise correlations between mean gFA derived from one‐directional voxels and motor behavior for each corticospinal sub‐tract reported for patients with persistent motor deficits. Bold font indicates significance after FDR‐correction.

Partial correlations assessing the association between premotor sub‐tract integrity and complex motor performance, while controlling for the influence of the M1 sub‐tract, were not significant after correction for multiple comparisons (all *p* > 0.05, FDR‐corrected). Similarly, the association between M1 sub‐tract anisotropy and complex motor skills did not remain significant when controlling for anisotropy of PMd, PMv, or SMA sub‐tracts (all *p* > 0.05, FDR‐corrected). These observations suggest a large degree of interdependence between M1 and premotor tract integrity for the execution of complex motor skills. In other words, the control of complex motor skills seems to critically rely on descending signals from both M1 and PMd, PMv, and SMA.

Of note, while complex motor skills assessed using the ARAT primarily reflect distal aspects of motor control captured by the subtests *pinch*, *grip*, and *grasp*, the *gross movement* subtest relies more strongly on proximal motor function. Given that tractwise anisotropy showed similar correlations with the four different subtests included in the ARAT, our results did not indicate a differential involvement of specific sub‐tracts in proximal versus distal motor control (see Table S4 for detailed results).

### Slicewise correlation analyses

**Figure 4:**
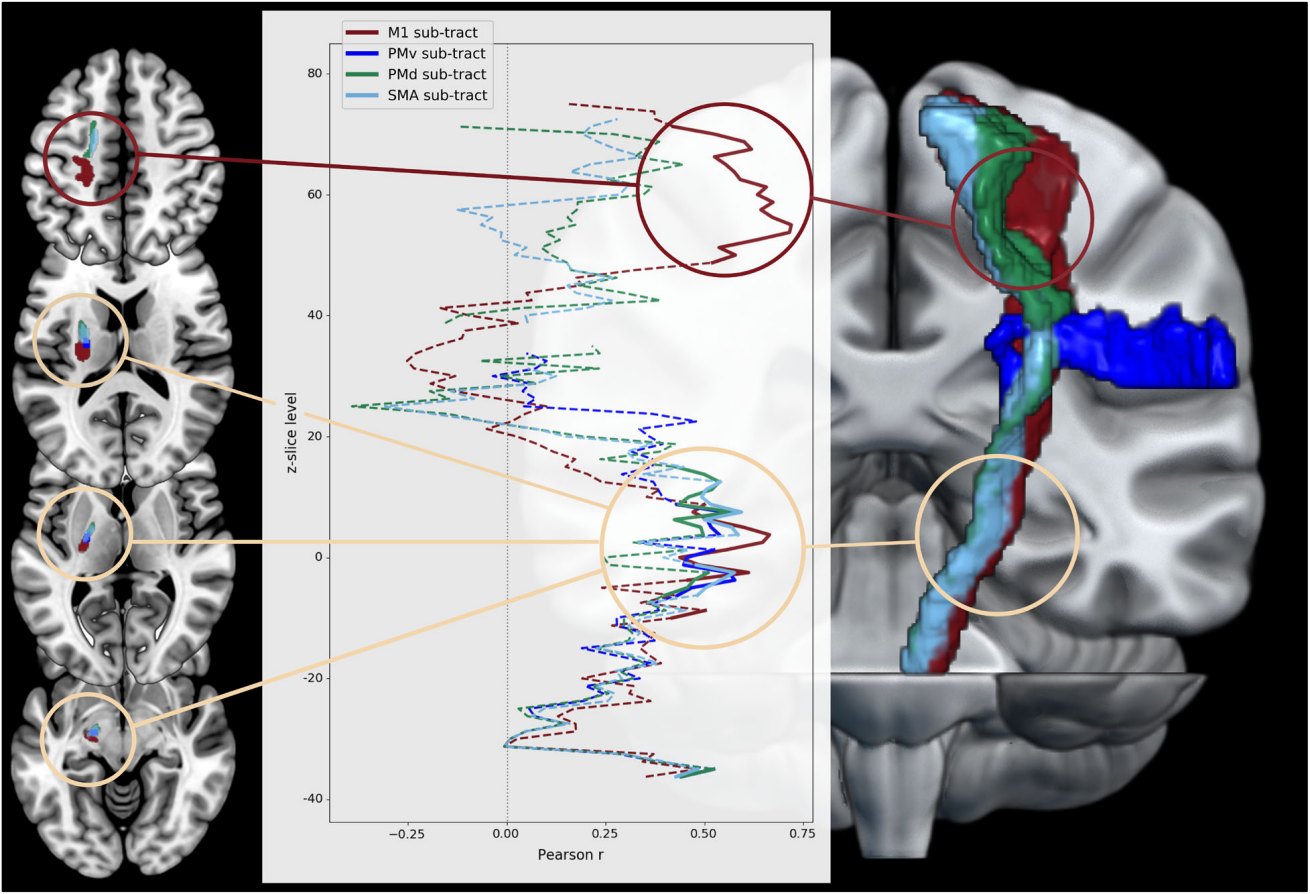
Slicewise correlations between mean anisotropy and complex motor control. For sub‐tracts originating from M1 (red), PMd (green), PMv (dark blue), and SMA (light blue), correlations between mean gFA and complex motor control were computed for each z‐slice. A cluster of significant correlations emerged at the level of the internal capsule ranging down to the mesencephalon consistently across all sub‐tracts. Only the M1 sub‐tract showed an additional section of significant correlations closer to the cortex in line with a potential cortical remapping. M1, primary motor cortex; PMd, dorsal premotor cortex; PMv, ventral premotor cortex; SMA, supplementary motor area.

To localize sections that were most indicative of motor performance for each corticospinal sub‐tract, we computed slicewise correlations between anisotropy derived from one‐directional voxels and motor performance. Premotor sub‐tracts originating from PMd, PMv, and SMA showed a consistent pattern of correlations with complex motor skills for the CST section extending from the internal capsule down to the mesencephalon (MNI z‐levels −6 to 15). The M1 sub‐tract featured a highly similar pattern for correlations with basal and complex motor skills (MNI z‐level −10 to 9). Of note, an additional section of significant correlations was observed for the M1 sub‐tract closer to the cortex (MNI z‐levels 49–72; Fig. 4).

## Discussion

Premotor areas are considered key players facilitating motor network reorganization after stroke.^4^ For example, fMRI studies have frequently reported increased activation of premotor areas during movements of the paretic hand^5^ as well as correlations between motor impairment and resting‐state or task‐related connectivity within the core motor network including M1, SMA, PMv, and PMd.^40^ Thus, motor recovery may arise from functional reorganization of cortico–cortical interactions between premotor areas and M1 facilitated by dense cortico–cortical fibers linking these regions. However, the aforementioned cortical motor areas also feature direct projections to the spinal level.^3^, ^22^, ^41^ Therefore, the question arises whether premotor areas facilitate post‐stroke motor control not only via well‐established cortico–cortical connections but also using their descending CST projections to the spinal level.

### Functional role of premotor areas in post‐stroke reorganization

In accordance with the notion that descending output from premotor regions is behaviorally relevant after stroke, previous studies have linked the integrity of descending premotor tracts to motor performance.^8^, ^9^, ^10^, ^11^, ^12^, ^13^ However, to date, studies do not converge toward a clear pattern regarding the functional relevance of specific sub‐tracts. Our findings offer a possible explanation for the somewhat contradictory results of previous publications as they revealed a dissociation of basal and complex motor control: a patient's ability to move the upper limb at a single joint as measured by the MI‐arm score was exclusively correlated with M1 sub‐tract integrity across the present cohort. Conversely, the successful performance of complex motor tasks was associated with sub‐tracts descending from M1, PMd, PMv, and SMA (Fig. 3). Of note, this distinction was attenuated in patients with persistent motor deficits.

Given that the integrity of the M1 sub‐tract was associated with both basal and complex motor performance, our findings highlight the preeminent role of motor output from M1, which constitutes a prerequisite for the elicitation of any form of muscle activity. Further support for the necessity of residual M1 output stems from the fact that the association between premotor sub‐tract anisotropy and complex motor skills depended on M1 sub‐tract integrity as suggested by the results of the partial correlation analyses. However, the fact that the correlation between M1 anisotropy and motor performance did not remain significant when controlling for premotor sub‐tract anisotropy highlights that M1 output alone is not sufficient for the execution of sophisticated movements of the affected arm and hand. Instead, motor commands seem to be shaped by descending signals originating from PMv, PMd, and SMA. Considering the pivotal role of PMv and PMd for reaching and grasping movements,^28^ the reliance on descending PMv and PMd output in post‐stroke motor control is well in line with their physiological roles in the control of hand movements. For the somewhat unanticipated yet strong correlation between complex motor performance and anisotropy of the SMA sub‐tract, three possible explanations should be considered. First, the functional role of the SMA including the performance of sequential movements and movement initiation might be instrumental for a wider range of tasks than initially assumed. Second, considering the significant association between M1‐premotor effective connectivity and motor impairment after stroke,^6^, ^40^ premotor areas including the SMA might serve as a “relay station” for M1 output. Following this logic, the SMA may receive motor commands from M1 via its cortico–cortical connections, thereby offering an alternative route to bypass damaged descending M1 fibers. Third, the functional distinction of premotor areas may partially macerate as a feature of cerebral reorganization after stroke. This would allow the intact premotor areas to flexibly compensate for functions of lesioned areas and their output tracts. Assuming this amount of flexibility, the functional role of a given premotor area may more heavily depend on the effects of the lesion than its physiological role in motor control in the healthy brain.

### Compensation via premotor areas in patients with persistent deficits

The motor network of severely affected patients has been shown to undergo more extensive reorganization processes.^42^ Hence, premotor CST fibers may play a distinct and potentially more important role in the reorganized brain of such severely affected patients. In line with this notion, anisotropy derived from premotor sub‐tracts originating from PMd, PMv, and SMA was linked to the MI‐arm score in a subsample of patients with persistent motor deficits (Table 2). Thus, in more severely affected patients with persistent motor deficits, premotor pathways seem to also support basal individual limb movements. From a mechanistic perspective, this may be interpreted as a difference in cortico–cortical interactions: Basal motor commands that would normally be transmitted via the M1 sub‐tract might be relayed from M1 to a premotor region in order to reach the spinal level via corticospinal premotor fibers. This view nicely matches previous reports of a correlation between basal motor performance and anisotropy of the cortical PMv‐M1 connection in patients with pronounced CST damage,^43^ thus suggesting an increased reliance on premotor areas via cortico–cortical connections following severe damage to descending M1 fibers. Hence, extensive damage to the M1 sub‐tract might cause a shift in basal motor control from M1 toward premotor areas at the cortical level to capitalize on intact or less affected CST fibers emerging from these premotor areas.

### Relevant CST sections

While lower CST sections are thought to be a valid indicator of Wallerian degeneration, CST fibers close to the cortex may also be subject to or contribute to cortical reorganization. Slicewise correlations between anisotropy and complex motor performance along the z‐axis of each tract suggested that the CST section ranging from the internal capsule down to the mesencephalon emerged as the most important segment across all tracts (Fig. 4). Notably, these particular sections are known to contain densely packed descending fibers^44^ and to be indicative of motor performance after stroke.^39^, ^45^ Of note, the correlation between motor control and the M1 sub‐tract was also driven by z‐slices closer to the cortex (Fig. 4). A possible explanation for this finding might derive from cortical remapping of M1 motor functions from damaged to intact cortical tissue.^42^, ^46^ In particular, axonal sprouting may result in novel efferent connections from remapped cortical areas to the CST. Alternatively, this observation may reflect the premorbid level of structural connectivity: Patients with less aging‐related atrophy may be able to draw on the structural reserve of descending and cortico–cortical motor network connections. While these mechanistic interpretations remain speculative, the additional section of relevant z‐slices closer to the cortex underlines the importance of considering voxels from the entire length of the CST, which constitutes an important difference from previous ROI‐based studies.^9^, ^11^

### Limitations and future directions

Given that complex motor behavior correlated with sub‐tracts originating from SMA, PMv, PMd, and M1, one might assume that a potential overlap between different sub‐tracts biased these correlations. However, the relatively small number of overlapping voxels across sub‐tracts renders a considerable sampling bias unlikely. Moreover, no association was observed between motor performance and anisotropy extracted from corticospinal sub‐tracts originating from preSMA or S1, corroborating the specificity of our findings. In particular, the preSMA has rich connections to regions of the prefrontal cortex and receives inputs from non‐motor areas of the dentate nucleus and globus pallidus,^47^ underlining its functional role as a prefrontal rather than a premotor area. Another limitation pertains to the relatively small sample size of our cohort. While the present sample size of 25 patients is not unusual for imaging studies in stroke patients, it may limit statistical power, especially regarding the subgroup analyses for patients with persistent deficits. Moreover, while the dMRI approach used in the present study is well suited to estimate tract integrity as reflected by tractwise anisotropy, it does not differentiate the physiological underpinnings that might cause the observed levels of anisotropy. Conceptually, a combination of factors including secondary degeneration processes such as anterograde or retrograde degeneration, as well as the premorbid level of structural connectivity is reflected by post‐stroke anisotropy. Disentangling the effects of secondary degeneration and structural reserve in future studies based on longitudinal data might hence offer valuable mechanistic insights. Finally, the present tract templates did not include fibers originating from cingulate motor areas.^48^ Addressing the compensatory potential of these additional corticospinal sub‐tracts constitutes a promising future endeavor.

## Conclusion

We here demonstrate distinct functional roles of corticospinal sub‐tracts originating from M1 and those originating from premotor areas for basal motor performance and complex motor skills after stroke. While descending signals from M1 were vital for both basal and complex movements, output from SMA, PMv, and PMd were relevant for the execution of complex motor skills in line with their physiological roles in motor control. However, premotor sub‐tracts showed an additional association with basal motor performance in patients with persistent motor deficits, emphasizing their functional flexibility in more severely affected patients. Thus, our current findings highlight the flexibility of premotor areas to adopt new functional roles during poststroke reorganization and suggest a functional relay of motor commands via descending premotor tracts after damage to M1 fibers. In conclusion, our results underline the importance of corticospinal motor signals from premotor areas for stroke recovery and propagate the focus on premotor areas for therapeutic interventions.

## Author Contributions

T.P., C.G., S.T.G., G.R.F., and L.J.V. contributed to the conception and design of the study. T.P., M.C., L.H., V.M.W., C.T., and L.J.V. contributed to the acquisition and analysis of data. T.P., S.T.G., and L.J.V. contributed to drafting the text or preparing the figures.

## Conflict of Interest

None.

## Supporting information


Tables S1–S4.

## Data Availability

Data are available from the corresponding author upon reasonable request.
