# Supplementary material for: Corticospinal premotor fibers facilitate complex motor control after stroke
Source: Ann Clin Transl Neurol. 2024 Jul 28;11(9):2439–49. doi: 10.1002/acn3.52159 (PMC11537136; doi:10.1002/acn3.52159)
Supplement: Supplementary file 1 — Tables S1–S4. [file ACN3-11-2439-s001.pdf]

Supplement

Supplementary Tables

Supplementary Table 1: Demographic and clinical patient information. *Abbreviations: ACA = anterior cerebral artery, ARAT = Action Research Arm Test, f = female, l = left, m = male, MCA = middle cerebral artery, MI = motricity index, PCA = posterior cerebral artery, r = right*

subject	sex	age	affected hemisphere	ARAT	MI-arm	lesion location	lesion volume (mm³)
1	m	70	l	19	65	MCA (subcortical)	1,211
2	f	84	r	57	83	Brainstem	582
3	m	66	r	38	76	MCA (subcortical)	11,559
4	f	74	r	56	91	MCA (cortical)	34,402
5	m	64	l	57	99	ACA/MCA (subcortical)	39,004
6	m	60	l	35	92	PCA (subcortical)	1,748
7	f	80	l	32	77	MCA (subcortical)	1,988
8	f	50	r	57	76	MCA (subcortical)	7,645
9	m	64	r	49	91	MCA (subcortical)	1,156
10	f	81	l	56	76	Brainstem	37
11	m	55	r	57	91	MCA (subcortical)	25,410
12	m	58	l	57	99	MCA (subcortical)	1,068
13	m	81	l	55	92	PCA (subcortical)	1,475
14	m	46	r	57	99	MCA (subcortical)	1,072
15	m	54	r	57	99	Brainstem	1,195
16	m	55	r	53	99	MCA (subcortical)	4,242
17	m	56	l	55	92	MCA (subcortical)	38,955
18	m	82	l	44	83	Brainstem	354
19	m	83	r	37	84	Brainstem	1,598
20	m	62	l	57	99	MCA (subcortical)	725
21	m	68	l	57	99	MCA (subcortical)	4,283
22	m	65	l	0	34	MCA (subcortical)	6,104
23	m	82	r	57	99	MCA (cortical)	37,850
24	m	68	l	57	99	MCA (sub- & cortical)	12,102
25	m	59	r	57	99	MCA (subcortical)	51,688

Supplementary Table 2: Tractwise anisotropy per subject. Mean gFA was derived for each sub-tract by averaging across all one-directional voxels included in the respective tract mask.

subject	M1	PMd	PMv	SMA	S1	preSMA
1	0.1015	0.0851	0.0870	0.0878	0.1047	0.0899
2	0.1163	0.1140	0.1162	0.1138	0.1182	0.1138
3	0.0943	0.1028	0.1008	0.0962	0.0968	0.1005
4	0.1208	0.1144	0.1107	0.1062	0.1166	0.1100
5	0.1124	0.0861	0.1045	0.0941	0.1157	0.0873
6	0.1022	0.0933	0.0980	0.0981	0.1045	0.0988
7	0.1119	0.1078	0.1002	0.1121	0.1159	0.1144
8	0.0985	0.1113	0.1070	0.1083	0.0928	0.1090
9	0.1217	0.1077	0.1140	0.1075	0.1184	0.1023
10	0.1134	0.1026	0.1099	0.1082	0.1207	0.1072
11	0.1140	0.1036	0.1023	0.1022	0.1122	0.0950
12	0.1259	0.1074	0.1174	0.1115	0.1309	0.1075
13	0.1250	0.1217	0.1234	0.1232	0.1271	0.1225
14	0.1161	0.1034	0.0985	0.1004	0.1077	0.0998
15	0.1028	0.1037	0.0966	0.0991	0.1010	0.1025
16	0.1109	0.1059	0.1012	0.1068	0.1050	0.1056
17	0.1181	0.0982	0.0968	0.1097	0.1160	0.0965
18	0.1157	0.1026	0.1056	0.1085	0.1193	0.1071
19	0.1154	0.1211	0.1087	0.1131	0.1115	0.1097
20	0.1167	0.1005	0.1060	0.1026	0.1223	0.1082
21	0.1183	0.0939	0.0971	0.0943	0.1173	0.0939
22	0.0988	0.0815	0.0895	0.0857	0.1092	0.0875
23	0.1250	0.1137	0.1067	0.1142	0.1220	0.0935
24	0.1189	0.1076	0.1099	0.1109	0.1213	0.1068
25	0.1102	0.0963	0.0918	0.0909	0.1045	0.0843

Supplementary Table 3: Non-parametric statistics. To ensure that the results were independent of specific distribution assumptions, we performed non-parametric permutation testing. Specifically, we computed permutation tests by randomly re-assigning motor score ~ anisotropy associations 1000 times across subjects and deriving a correlation coefficient for each of those random allocations, thereby creating a random sample-based distribution of correlation coefficients. The permutation-based p-value was then computed by dividing the number of correlation coefficients in the random sample-based distribution greater than the original correlation coefficient r by the number of correlation coefficients derived from re-sampling. Of note, the resulting permutation tests were perfectly in line with the results derived from parametric tests, thus corroborating our interpretations. Bold font indicates significance after FDR-correction.

DV	predictor	Pearson r	Pearson p	Pearson p (FDR)	permuted p	permuted p (FDR)
**ARAT**	**M1**	**0.548**	**0.005**	**0.027**	**0.003**	**0.009**
**ARAT**	**PMd**	**0.465**	**0.019**	**0.039**	**0.013**	**0.026**
**ARAT**	**PMv**	**0.501**	**0.011**	**0.032**	**0.001**	**0.006**
ARAT	S1	0.276	0.181	0.218	0.105	0.126
**ARAT**	**SMA**	**0.452**	**0.023**	**0.035**	**0.020**	**0.030**
ARAT	preSMA	0.250	0.228	0.228	0.110	0.110
**MI-arm**	**M1**	**0.562**	**0.003**	**0.021**	**0.005**	**0.030**
MI-arm	PMd	0.340	0.097	0.290	0.064	0.096
MI-arm	PMv	0.321	0.118	0.177	0.056	0.168
MI-arm	S1	0.271	0.190	0.228	0.100	0.120
MI-arm	SMA	0.327	0.111	0.221	0.061	0.122
MI-arm	preSMA	0.116	0.580	0.580	0.304	0.304

Supplementary Table 4: Association between tractwise anisotropy and ARAT sub-tests. To test whether anisotropy of certain corticospinal sub-tracts was more closely related to proximal or distal aspects of motor control, we computed correlations between tractwise anisotropy and the individual ARAT sub-tests (grasp, gross, grip, and pinch). The results depicted below indicate that each sub-tract was involved in both aspects of proximal and distal motor control.

ARAT	sub-tract	Pearson r	Pearson p	Pearson p (FDR)	permuted p	permuted p (FDR)
**grasp**	**M1**	**0.501**	**0.011**	**0.043**	**0.007**	**0.016**
**gross**	**M1**	**0.488**	**0.013**	**0.035**	**0.009**	**0.018**
**grip**	**M1**	**0.538**	**0.006**	**0.044**	**0.005**	**0.016**
**pinch**	**M1**	**0.571**	**0.003**	**0.046**	**0.001**	**0.016**
**grasp**	**PMd**	**0.440**	**0.028**	**0.034**	**0.018**	**0.022**
**gross**	**PMd**	**0.394**	**0.051**	**0.055**	**0.029**	**0.029**
**grip**	**PMd**	**0.451**	**0.024**	**0.034**	**0.013**	**0.021**
**pinch**	**PMd**	**0.482**	**0.015**	**0.033**	**0.013**	**0.021**
**grasp**	**PMv**	**0.462**	**0.020**	**0.040**	**0.004**	**0.018**
**gross**	**PMv**	**0.460**	**0.021**	**0.037**	**0.006**	**0.016**
**grip**	**PMv**	**0.496**	**0.012**	**0.038**	**0.004**	**0.018**
**pinch**	**PMv**	**0.511**	**0.009**	**0.048**	**0.002**	**0.016**
**grasp**	**SMA**	**0.431**	**0.031**	**0.036**	**0.015**	**0.020**
**gross**	**SMA**	**0.389**	**0.055**	**0.055**	**0.027**	**0.029**
**grip**	**SMA**	**0.445**	**0.026**	**0.034**	**0.013**	**0.021**
**pinch**	**SMA**	**0.460**	**0.021**	**0.033**	**0.021**	**0.024**
